# Smoking-induced changes in saliva composition and tongue coating

**DOI:** 10.7717/peerj.21228

**Published:** 2026-04-29

**Authors:** Nurul Asyikin Yahya, A’lia N. Muhammad, Nur Atikah Hashim, Mohammed Thoriq Abdul Razab

**Affiliations:** 1 Department of Family Oral Health, Universiti Kebangsaan Malaysia, Wilayah Persekutuan Kuala Lumpur, Malaysia; 2 Sandakan Dental Clinic, Sandakan, Sabah, Malaysia; 3 Changlun Dental Clinic, Changlun, Kedah, Malaysia; 4 Ar-Rizqin Sri Cemerlang Dental Clinic, Kota Bharu, Kelantan, Malaysia

**Keywords:** Smoking, Saliva buffering capacity, Tongue coating, Dental caries, Salivary pH, Saliva flow, Oral risk factors

## Abstract

**Background:**

Saliva parameters and tongue coating have global significance because they offer simple, non-invasive indicators of oral and systemic health, helping clinicians and researchers detect disease risk, monitor wellness, and improve early diagnostic strategies across diverse populations.

**Objective:**

This study aimed to investigate the effects of smoking on salivary flow rate, pH, buffering capacity, and tongue coating characteristics among adult smokers and nonsmokers.

**Methods:**

A cross-sectional study was designed to compare salivary parameters and tongue coating between smokers and nonsmokers. A purposive sampling method was used. The data collected included patients’ resting and stimulated saliva pH, saliva buffering capacity, and tongue coating for both smokers and nonsmokers. The inclusion criteria required the absence of systemic illness and medication that affected saliva flow.

**Results:**

A total of 78 participants were assessed for unstimulated and stimulated saliva flow, salivary pH, buffering capacity, and tongue coating (thickness, area, and colour). Smokers exhibited significantly reduced buffering capacity (*p* = 0.002) and increased tongue coating thickness and distribution area (*p* = 0.008 and *p* = 0.000, respectively), though saliva flow and pH differences were not statistically significant.

**Conclusion:**

These findings indicate that smoking is associated with reduced salivary buffering capacity and increased accumulation of tongue coating. Smoking may therefore represent a modifiable behavioural factor influencing salivary defence mechanisms and oral ecological balance. Integrating tongue coating and saliva analysis into caries risk assessment protocols could support earlier identification of individuals at increased risk.

## Introduction

Saliva plays a vital role in maintaining oral homeostasis by providing lubrication, antimicrobial protection, and buffering capacity (Song, Chung & Kim, 2024). It neutralises bacterial acids, facilitates enamel remineralisation, and aids mechanical cleansing of food debris (Mamani-Cori et al., 2025). Cigarette smoking has been consistently linked to adverse changes in the oral environment, including reduced salivary gland function, altered biochemical composition, and microbial dysbiosis (Majid, 2024). Tobacco smoke generates reactive oxygen species and toxic compounds that impair mucosal immunity and salivary protein function, thereby weakening the oral cavity’s natural defences (Pandarathodiyil et al., 2021). Alterations in salivary quantity or quality can shift the balance toward demineralisation, favouring caries progression (Yahya, Segaran & Tai, 2024). Dental caries remains one of the most common chronic diseases globally, affecting over two billion people and imposing a significant burden on public health systems (World Health Organisation, 2015). Its multifactorial nature involves interactions among microbial biofilms, dietary sugars, host susceptibility, favourable salivary parameters, and lifestyle behaviours (Jakubovics et al., 2021).

Beyond saliva, the tongue dorsum represents an important yet underexplored ecological niche in oral health research (Zakis et al., 2024). Tongue coating, composed of desquamated epithelial cells, food debris, and microorganisms, serves as a reservoir for microbial colonisation and the production of volatile sulphur compounds (Li et al., 2021). Smokers typically exhibit thicker and more extensive tongue coatings, which may reflect increased epithelial keratinisation and reduced salivary clearance. A study (Ahmadi-Motamayel et al., 2016) reported visible coating in 100% of smokers compared with 35% of nonsmokers, while another study (Omar et al., 2015) found that nearly 59% of smokers displayed coating compared with only 12% of nonsmokers. This accumulation not only contributes to halitosis but may also facilitate the recolonisation of cariogenic bacteria on tooth surfaces. Suzuki et al. (2022) showed that smoking alters the salivary and tongue microbiota, increasing pathogenic species such as *Fusobacterium nucleatum* and *Porphyromonas gingivalis*. The interaction among smoking, salivary alterations, and tongue coating may therefore represent a synergistic mechanism that influences caries risk (Li et al., 2021). However, few studies have evaluated these factors concurrently. Traditional caries risk models primarily focus on bacterial load and dietary patterns, often overlooking host-mediated parameters and mucosal ecology (Preisser et al., 2012). Integrating salivary and tongue-based biomarkers could enhance predictive accuracy in caries risk assessment, particularly for individuals exposed to behavioural or environmental stressors such as smoking.

Therefore, this study aimed to investigate the effects of smoking on salivary flow rate, pH, buffering capacity, and tongue coating characteristics among adult smokers and nonsmokers. By examining these parameters together, the research seeks to identify early, biologically relevant markers of oral environmental imbalance and to explore their potential application in caries risk prediction and preventive oral healthcare strategies.

## Materials and Methods

### Study design and population

A cross-sectional study design was employed among adult smokers and nonsmokers attending the Dental Polyclinics at Universiti Kebangsaan Malaysia (UKM). The sample size for this study was calculated based on Rad et al. (2010), who reported xerostomia incidence of 39% among smokers and 12% among nonsmokers. The sample size was calculated utilising a sample size calculator (Bujang, 2021). The probability of type-I error (α) was set at 0.05, and the probability of type-II error (β) was set at 0.2. Thus, the total sample size needed was 80 subjects to have 80% power. Allowing 10% dropouts, a final total sample size of 88 subjects, with 44 subjects in each group of smokers and nonsmokers. Study participants were selected using purposive sampling, screened for inclusion and exclusion criteria, and divided into smokers and nonsmokers. The inclusion criteria required the absence of systemic illness and medication that affected saliva flow. Smokers had a history of cigarette use for over six months. Written consent was obtained before the study. Ethical approval was obtained from Universiti Kebangsaan Malaysia (UKM PPI/111/8/JEP2018-2809).

Participants were recruited using purposive sampling to ensure adequate representation of both smokers and nonsmokers within the clinical setting. This approach allowed targeted recruitment of individuals who met predefined eligibility criteria. However, as a non-probability sampling method, it may limit external validity and generalizability to the wider population.

### Saliva collection and analysis

During the study, four tests using a saliva kit (Yahya, Segaran & Tai, 2024) were conducted and recorded to assess participants’ salivary parameters: (1) testing of resting saliva flow; (2) testing of stimulated saliva flow; (3) pH measurement of stimulated saliva and (4) buffering capacity. Before the saliva sample collection, all participants were advised to: (1) Rinse their mouths with water several times at the beginning of the test; (2) Swallow any remaining saliva before taking the sample collection; (3) Avoid speaking or swallowing during the test.

Unstimulated and stimulated saliva were collected under standardised conditions. Flow rate was measured volumetrically. Stimulated saliva was collected after chewing paraffin wax for five minutes. Resting saliva was collected by asking the participant not to swallow their saliva for 1 min, allowing the accumulated saliva to drip into a disposable cup. For stimulated saliva, the participant was asked to chew paraffin wax to stimulate salivary secretion and spit into the disposable cup after 30 s. They are then asked to continue chewing for an additional 5 min, collecting all the saliva into the collection cup at regular intervals. The quantity of saliva was measured by checking the mL markings on the cup. Normal stimulated saliva flow rate may range from one mL/min to 1.6 mL/min.

Salivary pH was assessed using pH indicator strips. The pH was measured by placing a pH test strip in the collected saliva for 10 s, then checking and recording the strip’s colour. The coloured test strip was compared with the testing chart available in the saliva kit. A highly acidic saliva is indicated in red, showing a pH range of 5.0–5.8. Moderately acidic saliva is indicated by a yellow colour, with a pH range of 6.0–6.6. While a healthy saliva is shown in green, showing a pH range of 6.8–7.8.

For the buffering capacity test, a buffer test strip (included in the saliva kit) was used, placed on an absorbent tissue with the test side facing up. Using a pipette, enough saliva was drawn from the collection cup, and one drop was dispensed onto each of the three test pads. The strip was immediately rotated 90 degrees to absorb excess saliva onto the absorbent tissue, preventing the test pad from swelling and affecting test result accuracy. After 2 min, the test pads changed their colour, and the result was calculated (Table 1). Buffering capacity was evaluated using colourimetric test strips and categorised as very low (0–5), low (6–9), or standard/high (10–12) (Table 1).

**Table 1:** Scores and colour codes of saliva buffering ability (Yahya, Segaran & Tai, 2024).

Total scores	Buffering ability of saliva	Colour codes
0–5	Very low	Red
6–9	Low	Yellow
10–12	Normal/ High	Green

### Tongue coating assessment

**Figure 1:**
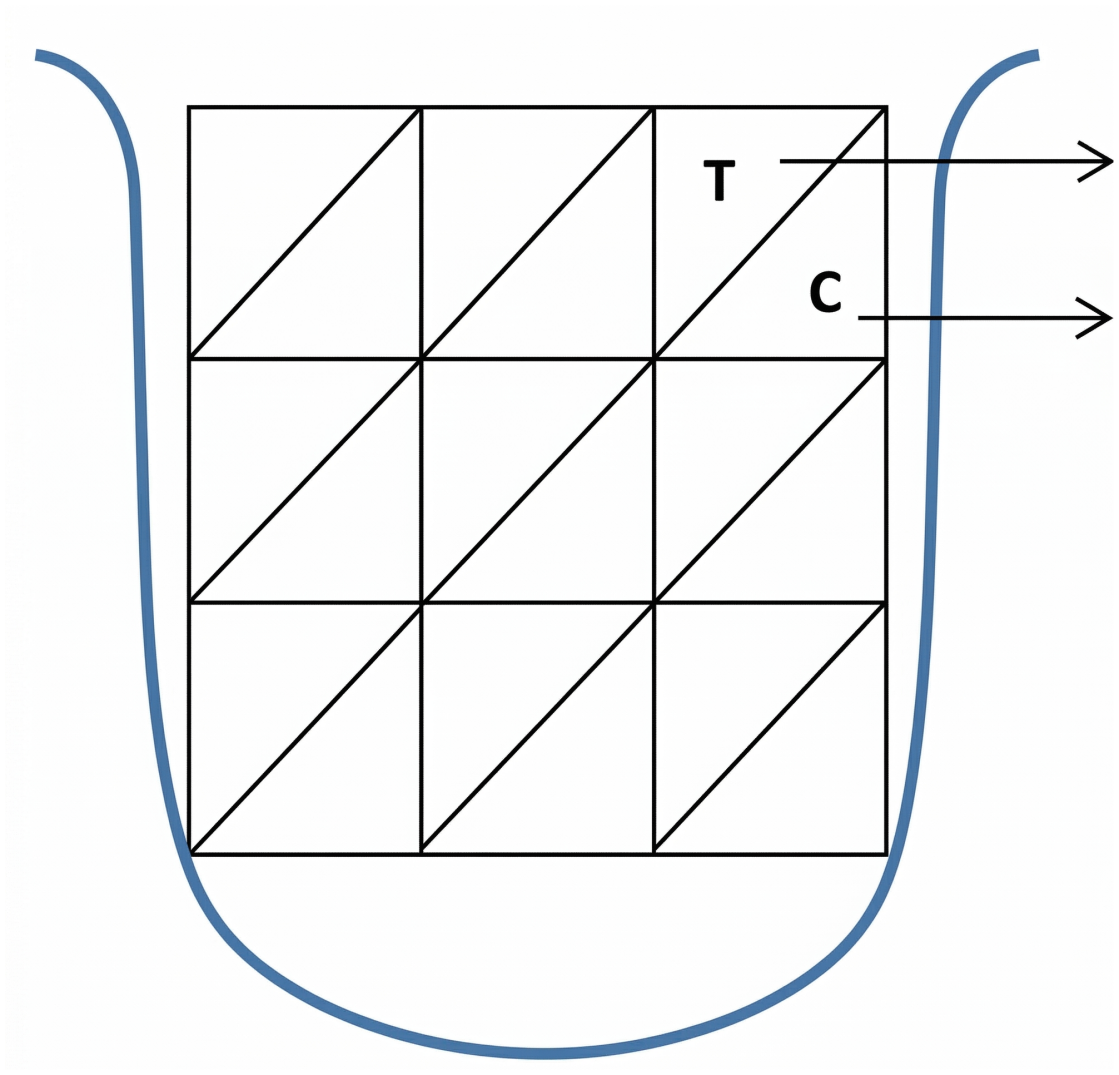
Tongue coating assessment on thickness (T) and colour (C).

Tongue coating was assessed using the Miyazaki Index (Miyazaki et al., 1995), which scores thickness (0–2), colour (0–3), and distribution area (0–3) across nine sections of the tongue dorsum (Fig. 1).

Three examiners conducted the examination, and an inter-rater reliability test was conducted among them prior to the study. The highest score per category was recorded for the participant. Inter-rater reliability (ICC = 0.889) was established before the examination, indicating good reliability (Koo & Li, 2016).

Tongue coating was assessed based on visual examination. Intraoral photos of the participant’s tongue were taken using smartphones for reference. The protocol for the tongue examination was as follows:

1. The tongue dorsum was visually divided into nine areas (Fig. 1), and the thickness of tongue coating within each area was recorded as either “0” for no coating, “1” for light coating, or “2” for thick coating. The colour of the tongue coating was also recorded by the nine divided areas of the tongue dorsum. If the colour of the tongue is pink, it is recorded as ‘0’, and if the colour is white, it is recorded as ‘1’, yellow as ‘2’ and brown as ‘3’.
2. For the thickness and colour of the tongue coating, the highest score out of nine areas was used as the final score for that individual. For the distribution area of tongue coating, the final score is determined by the total number of areas affected (Table 2).

### Smoking-related parameters

The Fagerström Test for Nicotine Dependence (FTND) and carbon monoxide analysis were conducted to confirm participants’ smoking status (Heatherton et al., 1991).

**Table 2:** Scores for intra-oral tongue-coating examination (Miyazaki et al., 1995).

Intra-oral tongue-coating examination	Scores
Amount/thickness of tongue coating	0 = no coating 1 = light coating 2 = severe coating
Distribution area	0 = Nonvisible 1 = Less than one-third of the tongue dorsum surface covered 2 = Less than two-thirds 3 = More than two-thirds
Colour of tongue coating	0 = Pink 1 = White 2 = Yellow 3 = Brown

A descriptive analysis was first performed, with categorical variables presented as frequencies and percentages and continuous variables as means and standard deviations. The chi-square test and the Mann–Whitney U test were used for comparative analyses between smokers and nonsmokers, with *p*-values < 0.05 considered significant. Given the modest sample size, analyses were limited to bivariate comparisons. Multivariate modelling was not performed; therefore, potential confounding variables, such as age and gender, were not adjusted for simultaneously.

## Results

Table 3 shows the socio-demographic distribution of the participants. The study included 38 smokers and 40 nonsmokers. Although the calculated minimum sample size was 88 participants (44 per group), the final analysed sample included 78 participants due to incomplete data and attrition. This slight reduction may have modestly affected statistical power, particularly for detecting small differences in salivary parameters. Among smokers, there were more males compared to nonsmokers (Table 3). Smokers and nonsmokers did not differ significantly in age or education level, though smoking participants were all male. In this study, the mean age of smokers was 31.89 years (SD = 12.33), and that of nonsmokers was 28.57 years (SD = 9.89).

Table 4 shows the distribution of salivary parameters among smokers and nonsmokers. No significant differences were observed in unstimulated or stimulated saliva flow or pH. However, buffering capacity was significantly lower among smokers (*p* = 0.002). Tongue coating was significantly more severe in smokers, as indicated by both thickness (*p* = 0.008) and area (*p* = 0.000), although colour differences were not statistically significant (*p* = 0.075). The quantity of saliva among smokers and nonsmokers showed no statistically significant difference for either resting saliva (*p* = 0.308) or stimulated saliva (*p* = 0.654). Other salivary parameters, including saliva pH, also showed insignificant differences between smokers and nonsmokers (*p* = 0.244). However, the buffering capacity among smokers and nonsmokers showed marked differences statistically (*p* = 0.002).

Table 5 shows the distribution of thickness, area and colour of tongue coating by smoking status. Severe tongue coating was reported in 54.3% of smokers and 20% of nonsmokers, with a statistically significant difference (*p* = 0.008). Moreover, approximately 37.3% of smokers and 18.7% of nonsmokers had more than two-thirds of the tongue coating area distributed in a statistically significant difference (*p* = 0.000). However, the colour of tongue coating between smokers and nonsmokers was not statistically significant (*p* = 0.075).

**Table 3:** Social characteristics of participants enrolled for the study (*N* = 78).

Characteristics	Smoking status		*p*-value
	Smokers (*n* = 38)	Nonsmokers (*n* = 40)	
Age (years) Mean ± SD	31.89 ± 12.33	28.57 ± 9.89	0.193
Gender			
Male, n (%)	38 (100)	16 (40.0)	0.000
Female, n (%)	0 (0)	24 (60.0)	
Ethnic			
Malay, n (%)	36 (94.7)	30 (75.0)	0.043
Indian, n (%)	0 (0)	3 (7.5)	
Chinese, n (%)	2 (5.3)	7 (17.5)	
Highest level of education			
Secondary education, n (%)	2 (6.7)	3 (10.3)	0.612
Tertiary education, n (%)	28 (93.3)	26 (89.7)	

**Table 4:** Distribution of salivary parameters among smokers and nonsmokers.

	Smoking status	*N*	Mean rank	Sum of ranks	*p*-value^a^
Rest Saliva	Smokers	38	36.87	1,401.00	0.308
	Non-smokers	40	42.00	1,680.00	
	Total	78			
Stimulated saliva	Smokers	38	38.33	1,456.50	0.654
	Non-smokers	40	40.61	1,624.50	
	Total	78			
Saliva pH	Smokers	38	36.47	1,386.00	0.244
	Non-smokers	40	42.38	1,695.00	
	Total	78			
Buffering capacity	Smokers	37	31.43	1,163.00	0.002
	Non-smokers	40	46.00	1,840.00	
	Total	77			

**Notes.**

a Mann–Whitney U test.

**Table 5:** Distribution of thickness, area and colour of tongue coating by smoking status.

		Smoking Status		Total	*p*-value^b^
		Smokers (38) n(%)	Non-smokers (40) n(%)		
Thickness of TC	No coating	1 (2.9)	3 (7.5)	4	0.008
	Light coating	15 (42.9)	29 (72.5)	39	
	Severe coating	19 (54.3)	8 (20.0)	21	
Area of TC	None visible	1 (2.9)	3 (7.5)	4	0.000
	<one third	0 (0)	10 (25.0)	10	
	1/3 − 2/3 of the tongue dorsum	6 (17.1)	13 (32.5)	17	
	>2/3 of the tongue dorsum	28 (37.3)	14 (18.7)	33	
Colour of TC	Pink	1 (2.9)	3 (7.5)	4	0.075
	White	21 (60.0)	31 (77.5)	44	
	Yellow	13 (37.1)	6 (15.0)	16	

**Notes.**

b Chi-Square test.

## Discussion

### Overview of findings

This study examined associations between cigarette smoking, salivary properties, and tongue coating characteristics. The findings demonstrated that smokers exhibited significantly lower salivary buffering capacity and greater tongue coating thickness and distribution area than nonsmokers. In contrast, salivary flow rate and pH did not differ significantly. These observations suggest that smoking is associated with alterations in salivary defence mechanisms and mucosal surface ecology, even among relatively young adults.

### Salivary flow rate and pH

No significant differences were observed in unstimulated and stimulated salivary flow rates between smokers and nonsmokers. These findings are consistent with previous reports (Khan, Javed & Ishaq, 2010; Ömeroğlu Şimşek et al., 2021), which found comparable salivary flow rates in smokers and nonsmokers, suggesting that long-term smoking may not immediately affect the salivary reflex mechanism. Similar results (Petrušić et al., 2015; Difaputra et al., 2025) have been attributed to the younger age of smokers and shorter exposure duration, factors likely applicable to the present cohort (mean age 31.89 ± 12.33 years).

The lack of a significant difference in salivary pH (*p* = 0.244) also aligns with several earlier investigations. Ahmadi-Motamayel et al. (2016) observed minimal differences between smokers (7.42) and nonsmokers (7.52), whereas other studies reported a slightly higher pH among smokers (Al-Weheb, 2005; Kakoei et al., 2021). However, Gupta et al. (2024) documented lower pH values in smokers, indicating variability across populations. Such inconsistencies may arise from differences in smoking intensity, sampling methods, or buffering composition—particularly bicarbonate ion secretion, which directly influences salivary pH.

Although no significant alterations were observed in this cohort, the biological plausibility of smoking-induced changes in salivary gland function remains strong. Recent evidence supports an association between chronic tobacco exposure, oxidative stress, and reduced salivary antioxidant capacity (Suzuki et al., 2022; Nigar et al., 2022). These effects may manifest progressively with longer smoking duration, warranting longitudinal evaluation in future studies.

### Salivary buffering capacity

The current study revealed a significant reduction in salivary buffering capacity among smokers, corroborating previous findings (Ahmadi-Motamayel et al., 2016; Khemiss, Ben Khelifa & Ben Saad, 2017). The reduced buffering capacity observed among smokers may reflect alterations in salivary biochemical defence mechanisms, potentially related to changes in bicarbonate and phosphate ion secretion or oxidative stress–related protein modification. Buffering capacity is a key protective property of saliva, essential for neutralising bacterial acids and maintaining pH stability on tooth surfaces (Matsuoka et al., 2025). Lower buffering potential facilitates acidogenic bacterial proliferation, promoting demineralisation and caries development (Ibrahim & Khalel, 2025).

### Tongue coating thickness and area

Smokers in this study exhibited significantly thicker and more extensive tongue coating than nonsmokers. This finding aligns with other studies (Ahmadi-Motamayel et al., 2016; Omar et al., 2015) that reported a markedly higher prevalence of tongue coating among smokers, as well as with observations that older age correlates with heavier coating. Smokers pose an increased risk of having Black Hairy Tongue compared to nonsmokers (Gurvits & Tan, 2014). The increased coating may result from epithelial keratinisation, altered desquamation, and reduced mucosal turnover, fostering anaerobic and acidogenic microbial accumulation (AlBeshri, 2025).

The presence of tongue coating has traditionally been linked to halitosis (Silva et al., 2020). Accumulated coating can serve as a bacterial reservoir, supporting cariogenic species such as *Streptococcus mutans* and *Lactobacillus* by retaining fermentable substrates (Zhang et al., 2021). Maintaining a healthy tongue microbiota may indirectly help prevent dental caries. Thus, the current findings suggest their potential relevance in caries risk models.

### Clinical and preventive implications

The combined reduction in salivary buffering capacity and increased tongue coating among smokers underscores smoking as a modifiable behavioural determinant of oral disease. These alterations may contribute to a more acidogenic and biofilm-supportive oral environment, which has been associated with increased caries risk (Maheswari et al., 2015). Integrating salivary buffering and tongue-coating evaluation into preventive protocols could enhance early detection of oral dysbiosis in smokers. From a clinical standpoint, incorporating these assessments into routine dental check-ups alongside targeted oral hygiene education and smoking cessation counselling may strengthen caries prevention strategies and improve long-term oral health outcomes.

### Limitations and future research

Several limitations should be considered when interpreting these findings. First, purposive sampling introduces potential selection bias and limits generalizability beyond the study population. Participants were recruited from a university clinical setting, which may not reflect broader community demographics.

Second, all smokers in the study were male, whereas the nonsmoker group included female participants. Sex-related differences in salivary flow rate and composition have been reported in previous literature. The gender imbalance in this sample may therefore have influenced observed differences and should be considered a potential confounding factor.

Third, although the age distributions between groups were not statistically different, multivariate analysis was not performed to adjust simultaneously for age, gender, or other possible confounders, such as oral hygiene practices, dietary habits, alcohol consumption, socioeconomic status, or smoking intensity (*e.g.*, pack-years). Future studies should incorporate multivariable regression models to strengthen causal inference and analytical robustness.

Fourth, salivary pH and buffering capacity were assessed using colourimetric test strips. While practical and clinically applicable, these methods are less precise than laboratory-based biochemical assays. Measurement variability may have reduced the sensitivity to detect subtle differences.

Finally, the cross-sectional design precludes establishing temporal or causal relationships between smoking exposure and alterations in salivary or tongue-coating parameters. Longitudinal studies are needed to evaluate progression over time and to determine whether these changes are reversible following smoking cessation.

## Conclusion

In conclusion, the present study demonstrates that smoking significantly reduces salivary buffering capacity and enhances tongue coating accumulation, potentially contributing to an environment conducive to dental caries. While salivary flow and pH were unaffected in this relatively young sample, the observed biochemical and surface changes highlight early indicators of oral ecological imbalance in smokers. These findings reinforce the importance of saliva-based diagnostics and tongue-coating assessments in future caries risk modelling and preventive dentistry frameworks.

##  Supplemental Information

10.7717/peerj.21228/supp-1 Supplemental Information 1 Saliva Tongue SPSS data

## Additional Information and Declarations

### Data Availability

The following information was supplied regarding data availability: The raw data is available in the Supplemental File.
